# Why Are African Researchers Left Behind in Global Scientific Publications? – A Viewpoint

**DOI:** 10.34172/ijhpm.2024.8149

**Published:** 2024-04-09

**Authors:** Juliet Nabyonga-Orem, James Avoka Asamani, Olushayo Olu

**Affiliations:** ^1^World Health Organization, Regional Office for Africa, Brazzaville, Congo.; ^2^Centre for Health Professions Education, Faculty of Health Sciences, NorthWest University, Potchefstroom, South Africa.

## Background

 Despite the many years of training and capacity building in health research, the low publication record by African scientists is a matter of concern. The modest improvements registered in the recent past, with a reported increase in Africa’s share of world publication output from 1.5% in 2005 to 3.20% in 2016,^1^ is nothing to celebrate. Africans’ contribution to the global share of 36 326 indexed publications on SARS-CoV-2/COVID-19 10 months into the pandemic was only 3.0%.^2^ A bibliometric analysis of scientific production on COVID-19 publications undertaken June 2020 shows a similar pattern. The best 10 African countries published fewer papers than what China alone published.^3^ Does the low productivity of African scientists partially explain the limited attention paid to Africa’s health challenges in the scientific literature? Several reviews bemoan the dearth of literature focusing on Africa. Naidoo et al^4^ also noted this seeming neglect regarding Africa’s pressing issues in COVID-19 related publications. In their review of COVID-19 related articles in 10 journals published between January 1, 2020 and September 30, 2020, only 3.9% of published articles had content relevant to Africa, only 3.2% of authors had an African primary affiliation and 66.1% of authors on African papers were not from Africa.

 The launch of the Sustainable Development Goals (SDGs) Agenda raised hope and galvanised commitment for all countries and populations. Some of the resonating phases were “of relevance to all countries irrespective of the level of development,” “reducing inequalities and leaving no one behind,” and “enhancing the capacity of developing countries to significantly increase the availability of high quality and timely data.” What did the launch of the SDG Agenda mean for African researchers?

 In this commentary, we highlight the low publication record in Africa and discuss the challenges faced by African scientists in producing and publishing high-quality articles. We conclude by proposing recommendations that can foster a comprehensive approach to building research capacity on the continent as a core component of the SDG agenda and specifically “leaving no one behind.”

## Low and Inequitable Investments in Health Research

 The investment by African governments in health research is regrettably very low compared to Europe and America, as shown in Table 1. A government per capita expenditure on research and development as low as $0.56 in Mauritania, and $1.56 in Gambia (2018) is a cause for concern. A survey of 39 countries of the World Health Organization (WHO) African Region in 2018 showed a similar pattern, with only 24 (61.5%) out of the 39 surveyed countries having a budget line for health research. Only 2 investing 2.0% of their national health budget on research and 1 country investing at least 5% of health sector development assistance on research.^5^ African governments have repeatedly bemoaned the reliance on donors to fund health research which they claim supports research that is not addressing priority evidence gaps, but what is needed is their commitment. Further, gaps in legislation and policy frameworks in Africa health research systems, and suboptimal institutional capacity have been documented.^6^ Kasprowicz et al^7^ have argued for an African-led health research capacity-strengthening approach with a special focus on African-based researchers who are well trained with clear career paths and strong collaboration. Unfortunately, with the current level of investment, this may remain a pipe dream. The low level of investments and weaknesses in health research systems need to be addressed to embrace the leave no one behind agenda enshrined in the SDGs, as it relates to African scientists.

**Table 1:** Government Expenditure on Research and Development

	**GERD as a Percentage of GDP**			**GERD Per Capita (in Current PPP$)**			**GDP Per Capita PPP Current figures (2019)**
	**2016**	**2017**	**2018**	**2016**	**2017**	**2018**	
Seychelles	0.22	-	-	103.10			30 898.20
Mauritius	-	0.37	0.35		78.46	78.74	23 841.00
South Africa	0.82	0.83	-	103.10	105.69		13 009.70
Egypt	0.71	0.68	0.72	79.30	74.83	84.22	12 261.20
Angola	0.03	-	-				6905.70
Côte d'Ivoire	0.07	-	-	3.32			5433.00
Mauritania	-	-	0.01			0.56	5416.90
Rwanda	0.65	-	-	12.23			2321.40
Gambia	-	-	0.07			1.56	2316.90
Ethiopia	-	0.27	-		5.55		2315.30
Burkina Faso	-	0.61	-		12.54		2270.40
Madagascar	0.01	0.01	-	0.20	0.20		1677.80
Chad	0.30	-	-	5.00			1646.40
Burundi	-	-	0.21			1.66	783.50
Africa (Sub-Saharan)	0.37	0.37	0.37	13.59	13.86	14.21	3885.50
Northern America	2.67	2.72	2.72	1517.6	1598.75	1676.02	63 766.00
Europe	1.83	1.86	1.89	638.48	685.76	721.76	46 466.30

Abbreviations: GDP, Gross domestic product; GERD, Gross domestic expenditure on R&D; PPP, purchasing power parity. Source of data: World Bank,^8^ and UNESCO Institute for Statistics.^9^

 Africa has not benefitted from available research grants in line with the global population and disease burden share. Further to the continent being disadvantaged compared to other regions of the world, additional inequitable intra-continental and diseases biased distribution is prevalent. In 2018, Africa received only 0.8% (583/76 435) of the grants provided by 11 funders. Further, there was inequitable distribution within Africa with South Africa receiving the highest number of grants (32.0%), followed by Kenya (17%) and Uganda (11%). There are countries that received only one grant (Sierra Leone, Liberia, Guinea, Gabon, Eswatini, Democratic Republic of the Congo, and Benin). Forty-three percent of the grants awarded to Africa addressed only 3 diseases, HIV, tuberculosis, and malaria.^10^

## How Resourced Are Health Research Systems in Africa?

 Despite years of investment, the capacity of health research systems in Africa is still low. Although efforts at the continental level have made modest improvements, gaps do persist. The endorsement of the health research strategy 2016-2015^11^ by ministers of health in the WHO African Region; the Africa Health Strategy 2016-2030^12^ by the African Union, the Health Research and Innovation Strategy for Africa 2018-2030 by the New Partnership for Africa’s Development,^13^ provided the impetus for a renewed focus on health research. Some improvements have been reported in strengthening ethical review, universities with training programs in health research, and countries that are regularly tracking expenditures on health research. However, gaps persist in governance and coordination of health research with 17 out of the 39 WHO African Region member states that were assessed in 2018 lacking legislation to regulate health research and 16 – lacking health research promoting unit within the Ministry of Health.^6^ Africa has only 20 Health researchers (in full-time equivalent) per million inhabitants compared to 239 in Europe (2021 figures).^10^

 Patchy and ad hoc investments may partially explain the persistent weaknesses in the health research systems. Strengthening health research capacity encompasses strengthening research governance, availing resources (human, financial, and infrastructure) in a sustainable manner, production and use of research and recognising the role of research in economic development. Attempts made have addressed only some of the components thus falling short of achieving overall desired goals. An evaluation of a 7-years program implemented by the West Africa Health Organisation that was aimed at enhancing the skills of health researchers through a series of post-graduate capacity building workshops concluded that; although the trainees developed protocols that were funded and implemented, there was a minimal influence on policy. The evaluation also concluded that the majority of the protocols were not published in scientific journals.^14^

 Significant and long-term investments in research by the European and Development Countries Clinical Trials Partnerships has registered successes but largely at the institutional level with only a modest impact on health research systems as a whole.^15,16^ The 15 years of investment by the United States National Institute of Health in sub-Saharan Africa, although successful, only focussed on strengthening the capacity for research ethics.^17^ Institutional capacity strengthening is arguably needed and beneficial, but we underscore the importance of a comprehensive approach to realise desired results. In as much as ethical capacity is a prerequisite to conducting sound research, Baluku et al bring to the fore the plight of early career researchers highlighting the prohibitive nature of the levied ethical review fees.^18^ Non-student early career researchers were paying up to 40% of their small research grants in ethical review fees.^18^

## Are Article Processing Charges s affordable to Africa-Based Researchers?

 Arguably, the SDGagenda is applicable to all populations but how about publishers of scientific journals who continue to charge unaffordable article processing charges (APCs) especially for African scientists? The high cost of publishing against a backdrop of hefty profits by the publishers has gone unabated despite repeated pleas.^19^ Weak health research systems plagued with suboptimal research infrastructure and a low number of poorly remunerated researchers, are known challenges facing Africa. Some may argue that these are major constraints impacting the volume of publications by African scientists. Although this is not entirely wrong, the issue of APCs, which the authors of this commentary have had to pay out of pocket several times is a major obstacle. Researchers from low-income countries have benefited from waivers provided by several journals which only partially address the challenge. The level of income of African governments does not necessarily translate into increased investment in health research. Levying APCs for African scientists seems like a deliberate effort to leave this population behind or impoverishing them.

 To further highlight their plight, the salaries of the most highly paid health professionals are shown in Table 2. We compare these to the APCs which range from £1500–4800 for BMJ Journals; US$ 860–4480 for Springer Nature Journals and US$ 1000–5200 for Willey Journals.

**Table 2:** Average Annual and Monthly Salary for Medical Specialists/Consultants in Selected Countries

**Country**	**Average Annual Income (US$)**	**Average Monthly Income (US$)**	**Source**
Ethiopia	5391	449	HLMA, 2020
Zimbabwe	14 431	1203	HLMA, 2021
Malawi	14 629	1219	SADC, 2019
Rwanda	18 316	1526	HLMA, 2019
Zambia	26 450	2204	NHWA, 2019
Lesotho	28 694	2391	HLMA, 2021
Sierra Leone	28 720	2393	HLMA, 2019
Ghana	29 821	2485	GHS, 2019
Eswatini	31 959	2663	SADC, 2019
Kenya	49 800	4150	HRH Strategy, 2019
Seychelles	63 303	5275	NHWA, 2020
Botswana	68 601	5717	SADC, 2019
Namibia	71 841	5987	HLMA, 2019
South Africa	90 476	7540	National HRH Strategy, 2020
*Mean*	*38* *745*	*3229*	
*Median*	*29* *271*	*2439*	
*Lowest*	*5391*	*449*	
*Highest*	*90* *476*	*7540*	

Abbreviations: HLMA, Health Labour Market Analysis; SADC, Southern Africa Development Community; NHWA, National Health Workforce Accounts; GHS, Ghana Health Services; HRH, Human Resource for Health.

 Comparing these figures shows that APC are not affordable, and one wonders what options are available for Africans. We conducted an online survey for researchers from African countries to which we received 25 responses. Seventy-one percent had published a paper in journals with impact factors, 88% had paid APC and 47% received full to partial waivers and 57% of these were not satisfied with the waivers they received. The prohibitive nature of APCs was highlighted by all respondents as one researcher stated that “*Publication fees are very prohibitive and stifle capacity of many potential authors from low- and middle-income countries.*” Indeed 82% of respondents considered publishing in a lesser-known journal because they were unable to afford APCs in their most preferred journals.

 The declaration by Nature (November 2020) that “*For €9500, Nature journals will now make your paper free to read*” was not welcome news for the African scientists. Much as we welcome the open access option as this would allow access to top-rated articles for a wider audience, there must be a balance between promoting the generation of evidence and improving access to the same, especially from African researchers. Indeed, the open access offer was received with mixed feelings. While some celebrate, researchers from Africa felt otherwise as indeed one of them stated that “*Alas! I think I will settle for just number of publications as opposed to the ranking of my articles, what else*?”

 Burgess-Jackson^20^ encourages scientists to publish in predatory journals, unfortunately, he presents an atypical case. He boasts of a publication record that is close to four decades, with the majority in top-rated journals, and he is already at the peak of his carrier. This notwithstanding, it is hard to find a balance in Burgess-Jackson’s arguments; on one hand resentful of the profiteering tendencies of top-rated journals, and on the other hand, proud of his publication track record in the same journals. Although one can accumulate articles through publishing in predatory journals, it does not offer the incentives that promote one’s career as such publications are not taken into consideration in determining promotions. Burgess-Jackson^20^ states that “*APCs are made known in advance […..], if you believe that a particular APC is excessive, you are free to go elsewhere.”* Where does this leave African scientists who have nowhere to go? The exorbitant profits made by some of the publishers (Elsevier US$ 2.58 billion (2019); Taylor & Francis US$ 330.4 million (2019); Wiley US$ 1.7 billion (2017)are disheartening in the face of an impoverished population of African scientists.

## Conclusion

 Despite the investments made into the African health research system, the research and publications output remain low on the continent due to both intrinsic and extrinsic factors. Unfortunately, the much-anticipated hope that locally generated evidence will inform the development of local solutions in African countries would remain a pipe dream, without building such research capacity. We therefore call for a comprehensive approach to building research capacity on the continent as a core component of the SDG Agenda and specifically “leaving no one behind.” We propose a few recommendations to achieve this objective. First is advocacy to African governments, Ministries of health and health decision-makers to increase funding to strengthen health system research and provide an enabling environment for health researchers on the continent. Researchers will only be productive working in strengthened health research systems that well-resourced, governed and capacitated. Partnerships in health research are beneficial, but these must be well negotiated and mutually beneficial. Second, we call on well-established publishers to create sister journals or special interest journals which publish evidence relevant to specific audiences at affordable costs to African authors. Third, donors, African governments, and academic and research institutions should collaborate to establish sustainable funds at a continental or global level that supports promising researchers to advance their careers and retain talent in Africa. However, this should be coupled with awareness raising to ensure that such opportunities are exploited. Fourth, African academic institutions in collaboration with Ministries of Health and international organizations should establish capacity-building programmes in scientific writing as both a pre-service and in-service initiative to enhance the research and publication skills of African researchers.

## Ethical issues

 Not applicable.

## Competing interests

 Authors declare that they have no competing interests.

## Disclaimer

 The views expressed in this article are views of the authors and do not represent views of the organisation they work for.
